# Structural Diversity, Fitness Cost, and Stability of a *Bla*_NDM-1_-Bearing Cointegrate Plasmid in *Klebsiella pneumoniae* and *Escherichia coli*

**DOI:** 10.3390/microorganisms9122435

**Published:** 2021-11-25

**Authors:** Ziyi Liu, Zhiqiang Wang, Xiaoyu Lu, Kai Peng, Sheng Chen, Susu He, Ruichao Li

**Affiliations:** 1 Jiangsu Co-Innovation Center for Prevention and Control of Important Animal Infectious Diseases and Zoonoses, College of Veterinary Medicine, Yangzhou University, Yangzhou 225009, China; lzy519461865@yeah.net (Z.L.); zqwang@yzu.edu.cn (Z.W.); Luxiaoyu2017happy@163.com (X.L.); pkai94@sina.com (K.P.); 2 Institute of Comparative Medicine, Yangzhou University, Yangzhou 225009, China; 3 Department of Infectious Diseases and Public Health, Jockey Club College of Veterinary Medicine and Life Sciences, City University of Hong Kong, Hong Kong 999077, China; shechen@cityu.edu.hk; 4 State Key Laboratory of Pharmaceutical Biotechnology, Medical School of Nanjing University, Nanjing 210093, China; susuhetian@nju.edu.cn; 5 Jiangsu Laboratory of Molecular Medicine, Medical School of Nanjing University, Nanjing 210093, China

**Keywords:** *bla*_NDM-1_, cointegrate plasmid, fitness cost, structural diversity, plasmid stability

## Abstract

Cointegrate/hybrid plasmids combine the genetic elements of two or more plasmids and generally carry abundant antimicrobial resistance determinants. Hence, the spread of cointegrate plasmids will accelerate the transmission of AMR genes. To evaluate the transmission risk caused by cointegrate plasmids, we investigated the structural diversity, fitness cost, and stability of a cointegrate plasmid in *Klebsiella pneumoniae* YZ6 and *Escherichia coli* EC600. The cointegrate plasmid pSL131_IncA/C_IncX3 was from a clinical *Salmonella* Lomita strain. After transferring the plasmid into *E. coli* EC600 by conjugation, we observed plasmids with different structures, including a full-length original plasmid and two truncated versions. By contrast, DNA fragment deletion and *bla*_CTX-M-14_ gene insertion in the plasmid were detected in a transconjugant derived from *K. pneumoniae* YZ6. These results suggest that the structure of the plasmid was unstable during conjugation. Furthermore, both the full-length plasmid in EC600 and the structurally reorganized plasmid in YZ6 imposed a fitness cost on the bacterial host and enhanced biofilm formation ability. Serial passaging in antibiotic-free medium resulted in a rapid decline of the plasmid in YZ6. However, the stability of the structurally reorganized plasmid in YZ6 was improved via serial passaging in antibiotic-containing medium. SNP calling revealed that mutations of the outer membrane porin may play an essential role in this process. These findings indicate that structural versatility could contribute to the dissemination of cointegrate plasmids. Although the plasmid incurred a fitness cost in other Enterobacteriaceae species, positive selection could alleviate the adverse effects.

## 1. Introduction

Plasmids, which are important drivers of bacterial evolution, code a wide range of traits that assist hosts in better adapting to complicated niches and stresses [1]. The function of plasmids in the dissemination of antimicrobial resistance (AMR) genes, facilitating the acquisition of multiple resistance genes by pathogens in a single-transfer event, is of great importance in various settings [2]. Generally, horizontally obtained AMR genes or multidrug-resistant (MDR) plasmids can impose an obvious fitness cost on the host [3], and are expected to be unstable during bacterial growth. Comparatively, plasmids can persist stably in bacterial populations in the absence of selection for plasmid-encoded traits, which is known as the plasmid paradox [4]. In laboratory settings, while plasmid persistence is usually observed, the level of selection required by plasmid persistence varies from no selection to different levels of antibiotic selection [5]. For example, *bla*_CTX-M-14_-carrying plasmid pCT, could persist and disseminate in the absence of antibiotic pressure [6]. Non-transmissible plasmid pNUK73, initially found in *P. aeruginosa* PAO1, was unstable, but compensatory adaptation increased its stability by alleviating the cost of plasmid carriage under a period of selection [1]. In addition, plasmid pKP33 was markedly less stable in naïve hosts (clinical strains that did not carry multidrug resistance plasmids) compared to the original host, but plasmid–host co-adaptation occurred in the presence of antibiotic selection, which improved its persistence after removal of selective pressure [7]. 

While the adaptation of small, non-conjugative plasmids is usually improved by selection pressure and compensatory adaptation, most AMR plasmids carry their own stability systems, such as toxin–antitoxin and partitioning systems, which confer stability in the bacteria by post-segregational killing or growth inhibition. Generally, the classical antitoxin forms a complex with the toxin to block its effects, and the TA complex is passed on to daughter cells. If the daughter cells do not inherit the plasmid, they are no longer able to produce more toxin or antitoxin molecules. With the degradation of unstable antitoxins, the toxins will exert their cytotoxic or growth inhibitory effects to ensure the maintenance of the plasmid in the cell population [8,9]. In addition, several hypotheses have been proposed to resolve plasmid persistence, such as host–plasmid co-adaptation, plasmid hitchhiking, cross-ecotype transfer, and high plasmid transfer rates; however, none of them could adequately clarify the plasmid paradox [5].

**Figure 1:**
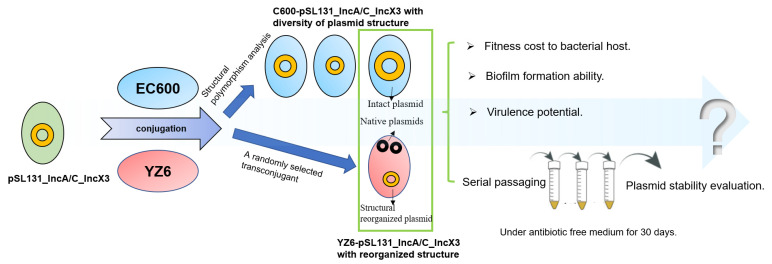
Schematic diagram of research process. A cointegrate plasmid (pSL131_IncA/C_IncX3) was transferred by conjugation into *Escherichia coli* EC600 and *Klebsiella pneumoniae* YZ6. Analysis of structural polymorphism was conducted after plasmid acquired by EC600. EC600 carrying intact plasmid and YZ6 carrying structural reorganized plasmid were subjected to fitness evaluation and serial passaging under antibiotic-free conditions for 30 days. Yellow circles in different sizes represented changes in cointegrate plasmid size during conjugation.

Plasmid fusion is increasingly detected in clinical and environmental bacterial isolates. Mobile elements are the main contributors to plasmid replicon fusion. Bacterial insertion sequences applying replicative transposition fuse transposon-bearing and target plasmids as a cointegrate [10]. In addition, mobile elements presenting as multiple copies can act as hot spots for homologous recombination. For instance, the presence of IS*26* in two plasmids of a carbapenem-resistant NDM-5-producing *E. coli* isolate led to plasmid fusion via homologous recombination [11]. Unlike traditional plasmids, due to the combination of genetic information of original plasmids, fusion/hybrid plasmids possess more AMR genes and insertion sequences with high plasticity. In recent years, the emergence of fusion/hybrid plasmids has been frequently reported, including *tet*(X4)-bearing plasmids [12], *mcr-1*-bearing plasmids [13,14], and other AMR genes carrying plasmids [15], causing serious public concern, as these plasmids play a crucial role in the dissemination of AMR genes. In this study, we investigated the structural diversity of a *bla*_NDM-1_-bearing cointegrate plasmid during conjugation and assessed the fitness cost and stability in order to systematically evaluate the transmission potential of this plasmid (Figure 1).

## 2. Materials and Methods

### 2.1. Bacterial Strains and Plasmids

The basic information of the cointegrate plasmid pSL131_IncA/C_IncX3 with different structures, including the presence of TA or partitioning systems, in this study is listed in Table S1. Strain YZ6 was a derivative of classical ST11 carbapenem-resistant *K. pneumoniae* HS11286 (accession number for the HS11286 chromosome is CP003200, and the six plasmids are CP003223 to CP003228) collected from sputum samples [16], and their characteristics are listed in Table S2. *Salmonella* Lomita SL131 was the natural host of the cointegrate plasmid pSL131_IncA/C_IncX3 [17], and rifampin-resistant *E. coli* EC600 was used as the recipient strain [18].

### 2.2. Filter Mating Assay and S1-PFGE

To investigate the structural diversity of pSL131_IncA/C_IncX3, conjugation assay was carried out between SL131 and EC600 using a filter mating method [19]. Transconjugants were selected on two types of antibiotics containing plates, one supplemented with meropenem and rifampicin (MR) and one supplemented with meropenem, tetracycline, and rifampicin (MTR). To characterize the profiles of cointegrate plasmids, SL131 and the corresponding transconjugants were digested with S1 nuclease (Takara, Osaka, Japan), followed by PFGE with the CHEF Mapper XA system (Bio-Rad, Hercules, CA, USA). *Salmonella* Braenderup H9812 standard strain restricted with XbaI was used as the molecular marker [20]. Conjugation assay was also performed between SL131 and YZ6. Transconjugants were screened by plates supplemented with hygromycin (200 mg/L), meropenem (2 mg/L), and tetracycline (16 mg/L).

### 2.3. Growth Curve Measurements

Overnight cultures of plasmid-free and plasmid-carrying strains were diluted to an optical density at 600 nm (OD600) of 0.05, and the diluents were grown at 37 ℃ for 12 h with vigorous aeration (200 rpm). The culture cell density was determined every hour and measured by a Thermo Scientific Multiskan FC Microplate photometer at OD600. All experiments were repeated 3 times.

### 2.4. Pairwise Competition Assay

Overnight cultures of plasmid-bearing strains and their plasmid-free isogenic ancestors were diluted to 0.5 McFarland standard and mixed at a ratio of 1:1 in 5 mL LB broth (Haibo Biotechnology Co., Ltd., Qingdao, China) [21]. Then, the mixtures were incubated at 37 °C for 72 h with shaking. Every 24 h, 5-μL cultures were reinoculated in 5 mL of fresh LB medium. The number of cells for each strain was determined by spreading serial 10-fold dilution onto LB agar plates with or without 2 mg/L of meropenem and 16 mg/L of tetracycline, and the relative fitness was calculated as follows: w = ln(NRt/NR0)/ln(NSt/NS0), where NR is the number of resistant clones and NS is the number of susceptible clones, with values below 1 indicating the fitness cost.

### 2.5. Galleria Mellonella Larval Infection Assay

For *Galleria mellonella* larval infection assay, about 300 mg of larvae was stored in a special box at 4 °C until use. Overnight cultures were washed and adjusted to 10^6^ CFU/mL using PBS. Ten larvae in each group were challenged with 10 μL of diluent, and PBS was used as the negative control. Infected larvae were incubated in sterilized Petri dishes at 37 °C for 72 h, and the survival rate was recorded every 24 h.

### 2.6. Biofilm Formation

Biofilm formation assay was conducted as previously described [22]. Overnight cultures were adjusted to a cell density equivalent to a 0.5 McFarland standard. Then 200 μL of each culture was transferred to a 96-well plate in triplicate. After being incubated at 37 °C for 2 days, the cultures were discarded. Each well was washed twice with 200 μL of PBS. The biofilms were fixed in methanol for 10 min. Subsequently, each well was stained with 1% crystal violet solution for 10 min and rinsed with PBS until colorless. Finally, biofilms were dissolved in 100 μL of 30% formic acid for 30 min, and biofilm formation was quantified by measuring the absorbance at OD590.

### 2.7. Plasmid Stability Experiments

First, the cointegrate plasmid-bearing EC600 and YZ6 were propagated by serial passaging for 30 days in antibiotic-free LB broth. Every 12 h, 5 μL of each culture was transferred to 5 mL of fresh LB broth. To evaluate the stability of the cointegrate plasmid, the fraction of plasmid-containing cells in population was calculated every 5 days by counting the number of colonies that grew on antibiotic-free and antibiotic-containing plates.

Subsequently, the cointegrate plasmid-carrying YZ6 strain was passaged for 30 days under meropenem and tetracycline pressure. The plasmid persistence was determined for 8 days as previously described with minor modification [23]. 

### 2.8. DNA Sequencing and Bioinformatics Analysis

The genomic DNA of the ancestral and three evolved strains was extracted using the TIANamp bacterial DNA kit (TianGen, Beijing, China) and subjected to short-read sequencing (2 × 150 bp) with the Illumina HiSeq 2500 platform. Short-read Illumina raw sequences of ancestral and evolved strains were separately assembled using SPAdes [24], and contigs less than 500 bp were discarded. SNP analysis was performed using Snippy (4.0.2) against the genome of the ancestral strain [25].

The plasmids of the three transconjugants of EC600, as well as pSL131_IncA/C_IncX3-133K and pSL131_IncA/C_IncX3-ev1, were extracted using a Qiagen Plasmid Midi-Kit (Qiagen, Hilden, Germany). Subsequently, the plasmids were sequenced with the Oxford Nanopore Technologies MinION long-read platform. The nanopore long-read MinION sequences of plasmids were subjected to de novo assembly with the Flye tool [26]. Among them, raw sequences of pSL131_IncA/C_IncX3-133kb less than 30 kb were discarded before the assembly. BRIG and Easyfig were used to describe the structural diversity of cointegrate plasmid during conjugation [27,28].

### 2.9. Statistics

GraphPad Prism (6.0.1) was used to compare growth curves, relative fitness, biofilm formation ability, survival rate, and plasmid stability. Differences in survival were compared using the log-rank (Mantel-Cox) test. Absorbance values for biofilm formation between strains with and without the cointegrate plasmid were compared using the *t*-test. Statistical significance was set at *p* < 0.05.

## 3. Results and Discussion

### 3.1. Structural Diversity of Cointegrate Plasmid pSL131_IncA/C_IncX3 in EC600 after Conjugation

Plasmid pSL131_IncA/C_IncX3 of *Salmonella* Lomita origin about 216 kb in size was a *bla*_NDM-1_-bearing cointegrate plasmid consisting of IncX3 and IncC plasmid backbones [17]. The *floR*, *tet*(A), *strAB*, *sul2*, *bla*_CMY-2_, *dfrA12*, *aadA2*, *sul1*, *aph(3′)-Ia*, and *mph*(A) genes were located on IncC plasmid backbone, while *bla*_NDM-1_ was detected on IncX3 plasmid backbone. In addition, it contained a type II TA system *relE-Xre*-like and partition module *parA*/*parB*, which are known to stabilize plasmid after replication and partitioning [29]. Our previous study revealed that the structure of the plasmid was prone to resolve single IncX3 plasmid during conjugation [17], yet the dynamic changes of the structure under the selection of other antibiotic combinations remained to be investigated. 

To probe the structural polymorphism of the plasmid during conjugation, a conjugation assay was performed using EC600 Rif^R^ as recipient strain. We randomly selected 11 transconjugants from MTR plates and 30 transconjugants from MR plates. Consistent with the previous study [17], a plasmid about 53 kb in size was detected in the 30 transconjugants from MR plates, suggesting that only the resolved IncX3 plasmid was present in them (Figure S1). By contrast, pSL131_IncA/C_IncX3 exhibited various sizes in the 11 transconjugants from MTR plates, which might be the consequence of plasmid reorganization (Figure S2). In general, some transconjugants from MR plates should be of MTR phenotype. However, the transconjugants from the two types of plates showed distinct differences. This was largely due to the selection-free IncC plasmid backbone triggering the resolution of fusion plasmid, and IncC plasmid was immediately discarded by the recipient strain during conjugation. To explore the underlying molecular mechanism of plasmid reorganization, three representative plasmids in transconjugants from MTR plates with different sizes were selected for nanopore sequencing. 

**Figure 2:**
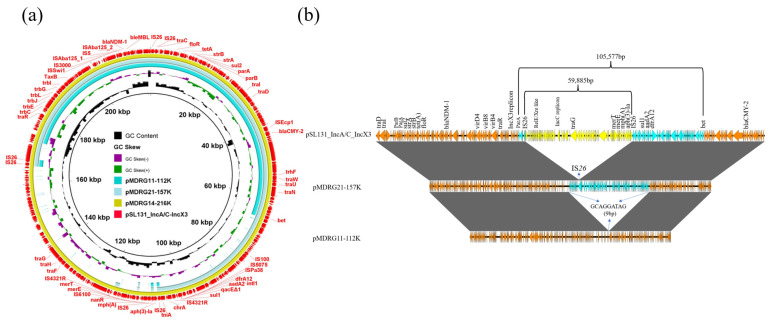
(**a**) Circular comparison of cointegrate plasmid pSL131_IncA/C_IncX3 with three plasmids from transconjugants of EC600 selected by MTR plates. Outermost circle with red arrows represents reference pSL131_IncA/C_IncX3. (**b**) Linear comparison of cointegrate plasmid pSL131_IncA/C_IncX3 with two structurally altered plasmids. Arrows marked in yellow indicate discarded regions from pSL131_IncA/C_IncX3 to pMDRG21-157K, and regions marked in yellow and blue represent discarded regions from pSL131_IncA/C_IncX3 to pMDRG11-112K.

Plasmid pMDRG14 shared a similar genetic structure with the original plasmid, indicating that the plasmid could transfer into the recipient strain with a full-length version (Figure 2a). Compared to the original plasmid, pMDRG21-157K discarded an approximately 59 kb region, including *aph(3)-Ia*, *mph*(A), heavy metal tolerance *mer* operon, *Rel-Xre*-like TA system, and IncC replicon. Thus, the plasmid belonged to IncX3 type, rather than the original multireplicon type. This event was mediated by IS*26*, which plays a critical role in the dissemination of antibiotic resistance genes and formation of complex antibiotic resistance regions in Gram-negative bacteria [30]. The last plasmid, pMDRG11-112K, deleted a 105 kb region, including the aforementioned region, as well as a 39 kb region containing an integron between DNA recombination gene *bet* and IS*26*, and a 7 kb region containing partitioning gene *parA* located between IS*26* and a hypothetical protein. The deletion leading to the plasmid formation was flanked by short sequence homologies (9 bp) on two genes with unknown function (Figure 2b). These findings indicate that the structure of pSL131_IncA/C_IncX3 was highly plastic and could be enriched under different antibiotic selection during conjugation. Conjugative events play an important role in the development of large plasmids, promoting plasmid evolution [31]. Considering that a limited number of transconjugants were selected and characterized, the possibility of other types of plasmid reorganizations could not be fully investigated. Nevertheless, our results at least highlight the high-plasticity trait of the plasmid, which may be regarded as the driving force in plasmid transmission and evolution.

### 3.2. Cointegrate Plasmid pSL131_IncA/C_IncX3 Experienced Structural Reorganization in K. pneumoniae YZ6 after Conjugation

To investigate the fitness cost of this cointegrate plasmid imposed on other Enterobacteriaceae hosts, the plasmid was further conjugated into *K. pneumoniae* YZ6 Hyg^R^. A transconjugant named YZ6-pSL131_IncA/C_IncX3-133K was randomly selected for Nanopore MinION long-read sequencing. It carried the cointegrate plasmid with 133,188 bp, in which two regions were discarded in the IncC plasmid backbone. One of the regions was 66 kb and consisted of various genes encoding plasmid conjugative transfer protein, *bla*_CMY-2_ gene, mobile elements, and hypothetical protein. The region was replaced by a gene that encoded the IS*3* family transposase from the chromosome of YZ6. The other region contained *dfrA12*, *aadA2*, *sul1*, *aph(3)-Ia*, and *mph*(A) genes and heavy metal tolerance *mer* operon, and it was flanked by 7 bp short sequence homologies. Moreover, the sequence of gene-encoding RHS repeat protein was partially lost, resulting in gene truncation. This deficiency was different from that in EC600, suggesting that the high variability of the plasmid structure may help the plasmid adapt to different host bacteria. 

**Figure 3:**
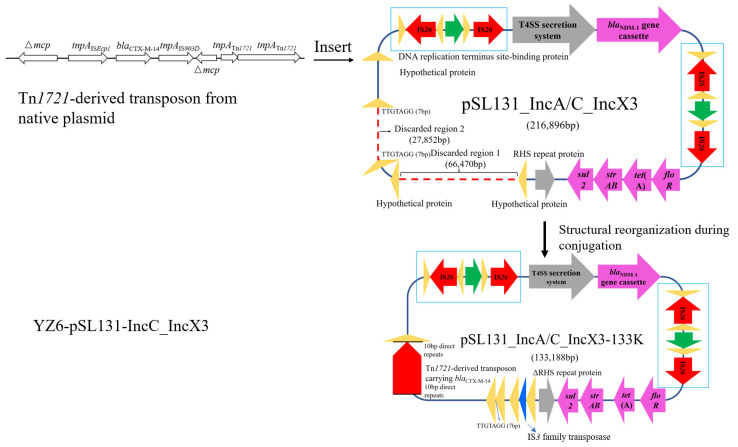
Schematic diagram of structural reorganization of pSL131_IncA/C_IncX3 after being conjugated into YZ6. Regions marked with dashed lines indicated discarded sequences; 66 kb was replaced by gene encoding IS*3* family transposase. Deletion of the 27 kb region was mediated by short sequence homologies. The *bla*_CTX-M-14_ gene from native plasmid was inserted into a gene-encoding DNA replication terminus site-binding protein mediated by Tn*1721*-derived transposon and left two 10 bp direct repeats.

In addition, the plasmid acquired a Tn*1721*-derived structure carrying *bla*_CTX-M-14_ from native plasmid pKPHS1 of YZ6 [16]. The Tn*1721*-derived structure inserted into gene encoding DNA replication terminus site-binding protein and generated a 10 bp direct repeat as the target site duplication upon mobile element insertion (Figure 3). After genetic recombination, the plasmid updated the profiles of AMR genes and insertion sequences, which raised concern about the emergence of novel plasmid. A recent study revealed that *bla*_CTX-M-27_ was able to transfer through transposition of a Tn*1721*-like structure between plasmids of *Salmonella* and *E. coli* [32]. Moreover, our findings prove that the transposon could transfer between different plasmids within bacteria. Tn*1721*-like transposons were found in numerous Gram-negative bacterial genomic or plasmid sequences [33]. Apart from *bla*_CTX-M_, Tn*1721*-like structure was also found to be associated with the dissemination of *fosA3*, *bla*_KPC-2_, and tetracycline resistance genes [34,35]. These findings indicate the wide distribution of the transposon among clinical settings, and it is necessary to continuously monitor the plasmid evolution events mediated by this transposon.

### 3.3. Cointegrate Plasmid pSL131_IncA/C_IncX3 Could Impose Different Levels of Fitness Cost in Klebsiella pneumoniae and E. coli

**Figure 4:**
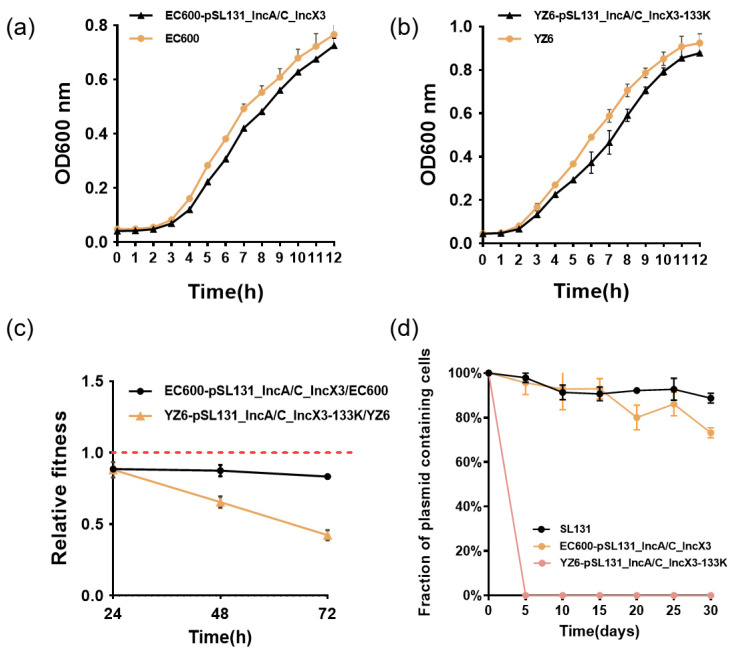
Initial fitness cost after acquisition of cointegrate plasmid in EC600 and YZ6 strains and plasmid persistence results of SL131, EC600-pSL131_IncA/C_IncX3, and YZ6-pSL131_IncA/C_IncX3-133K. (**a**,**b**) Growth curves of EC600 and EC600-pSL131_IncA/C_IncX3, YZ6 and YZ6-pSL131-IncA/C-IncX3-133K. (**c**) Relative fitness of two pSL131_IncA/C_IncX3 carrying strains. Relative fitness value less than 1 indicates fitness defect, and a value greater than 1 indicates fitness benefit. (**d**) Plasmid stability in bacterial population after serial passaging in antibiotic-free medium. Each point is the mean of three individual replicates, and error bars show standard deviation.

The transconjugant YZ6-pSL131_IncA/C_IncX3-133K and aforementioned MDRG14 (EC600-pSL131_IncA/C_IncX3) were subjected to fitness cost evaluation. According to the bacterial growth curves, we observed that the plasmid caused an obvious decrease in growth rate of EC600 and YZ6 (Figure 4a,b). Pairwise competitions were conducted between plasmid-bearing strains and their plasmid-free isogenic ancestors. The relative fitness of both YZ6 and EC600 was less than 1, suggesting that the plasmid imposed a fitness cost on EC600 and YZ6 (Figure 4c). Several studies have reported the fitness effects after acquisition of MDR plasmid on host bacteria. A *bla*_KPC-2_ carrying cointegrate plasmid pT18 was found to affect the growth condition of *E. coli* DH5α [36]. On the contrary, significantly increased fitness in *E. coli* DH5α was observed after co-introduction of two plasmids harboring *bla*_NDM-1_ and *bla*_OXA-232_ [37]. Our data suggest that the plasmid imposed an extra burden on both YZ6 and EC600, which partly hindered the prevalence of the plasmid. 

**Figure 5:**
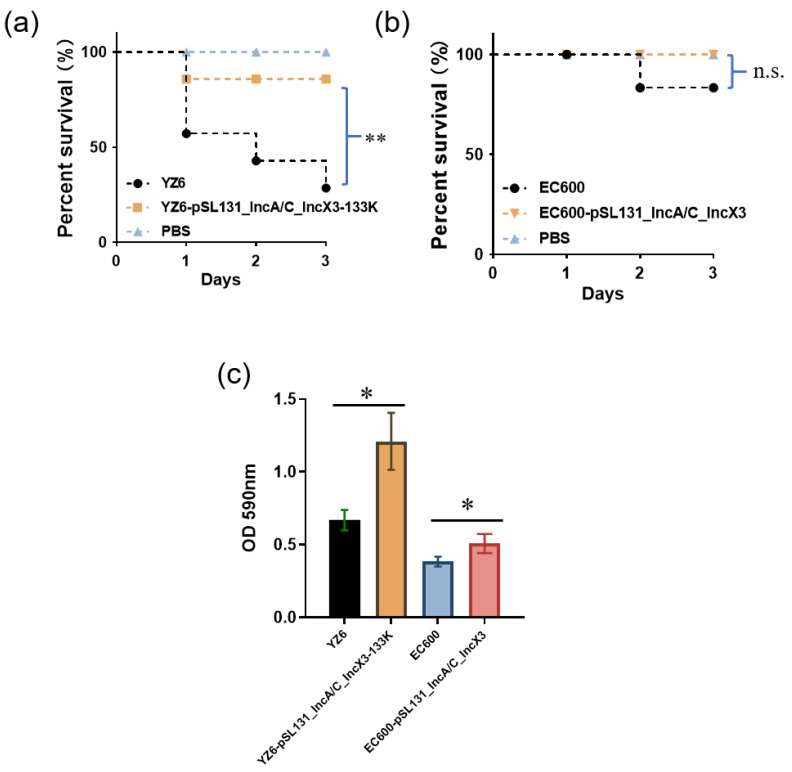
Results of virulence assay and biofilm formation ability of YZ6 and EC600, and their corresponding transconjugants. (**a**) Virulence potential of YZ6 and YZ6-pSL131_IncA/C_IncX3-133K in a *G. mellonella* infection model. ** *p* < 0.01. (**b**) Virulence potential of EC600 and EC600-pSL131_IncA/C_IncX3 in a *G. mellonella* infection model. n.s, no difference. (**c**) Biofilm formation ability of YZ6 and YZ6-pSL131_IncA/C_IncX3-133K, EC600 and EC600-pSL131_IncA/C_IncX3. * *p* < 0.05.

In addition, we compared the virulence and biofilm formation ability between the strains with and without the cointegrate plasmid. YZ6-pSL131_IncA/C_IncX3-133K showed decreased virulence compared to YZ6 (*p* < 0.01), which could be because the reduced fitness is expected to impair the bacterial virulence [38]. Nevertheless, the introduction of cointegrate plasmid did not affect the virulence of EC600 (n.s.). Biofilm formation assay revealed that both YZ6 and EC600 had significantly increased biofilm formation ability after plasmid acquisition (*p* < 0.5) (Figure 5). Biofilms play an important role in bacterial infection and the spread of AMR genes [39], especially in clinical settings, where they contribute to the expansion of various pathogens and AMR genes [40]. For instance, there was already evidence that the formation of biofilm in *Staphylococcus aureus* could promote the horizontal transfer of plasmid-borne AMR genes [41]. Thus, if the plasmid were widely distributed among clinical settings, it would inevitably complicate treatment and prevention measures.

### 3.4. Stability of Plasmids in YZ6, EC600, and Natural Host in Antibiotic-Free Medium

In order to evaluate the stability of pSL131_IncA/C_IncX3 in its natural host SL131, as well as in YZ6 and EC600, in the absence of antibiotics, we propagated the plasmid-harboring SL131, YZ6, and EC600 in antibiotic-free LB broth for 30 days. The plasmid could be stably maintained in its natural host and EC600 in at least 70% of cells. The persistence of the plasmid in the natural host was largely a consequence of the long-term co-evolution of host and plasmid [17]. However, the plasmid was extremely unstable in YZ6, since rapid decline of the plasmid was observed within 5 days (Figure 4d). Both partitioning systems and TA systems were observed in the reorganized plasmid pSL131_IncA/C_IncX3-133K (Table S1). However, they were unable to counteract the fitness cost imposed by plasmid in the host, resulting in plasmid loss during bacterial cell division. Furthermore, a recent study revealed that plasmid loss was determined by transcription–replication conflicts, and abiotic factors, such as temperature, should also be considered as factors influencing plasmid persistence [42], since the transcription–replication conflicts frequently arose following the acquisition of new genes (i.e., the insertion of mobile genetic elements could disrupt plasmid replication and thus lead to plasmid instability and extinction), and cold temperature appeared to be favorable for plasmid persistence.

### 3.5. Stability of Structural Deficiency Plasmid Was Improved in YZ6 under Positive Selection

Given that pSL131_IncA/C_IncX3-133K was unlikely to persist in *K. pneumoniae* YZ6 in antibiotic-free medium, we performed a serial passaging experiment in LB broth containing meropenem (2 mg/L) and tetracycline (16 mg/L) for 30 days. After evolution under positive selection, three randomly selected clones were subjected to persistence assay in antibiotic-free LB broth for 8 consecutive days. While the plasmid was initially unstable in host bacteria, it was improved after long-term evolution, as the plasmid-containing cells dominated the cells by more than 70% after eight days of antibiotic-free passaging (Figure S3). 

To investigate the potential mechanisms for the improved plasmid persistence, we performed whole-genome sequencing and analyzed the SNPs between ancestral and evolved strains. SNPs were located on chromosomes, and no SNPs were found in plasmids (Table S3). The chromosomal mutations of all evolved strains occurred in genes encoding hypothetical protein and outer membrane porin (OMP), and the SNP number of OMPs in YZ6-pSL131_IncA/C_IncX3-ev2 and YZ6-pSL131_IncA/C_IncX3-ev3 was higher than that in YZ6-pSL131_IncA/C_IncX3-ev1. Besides, no additional rearrangements were detected in evolved plasmid (Figure S4). 

Large conjugative plasmids have three evolutionary patterns to increase persistence: mutations to replication genes, mutations to the global transcriptional regulatory system of the host, and changes in the plasmid backbone (i.e., deletions of costly regions of plasmid and acquisition of a toxin-antitoxin transposon) [5]. Several studies have demonstrated that imposing positive selection on plasmids usually resulted in compensatory evolution via plasmid or host chromosome mutations that facilitated the plasmid’s persistence [43,44,45]. As previously mentioned, OMPs were strongly associated with antibiotic resistance, and the degree of expression was also related to the fitness cost of the bacteria [46]. Moreover, single-point mutation alone was sufficient to affect OMP function [47]. Thus, we hypothesized that the OMP mutations were likely to ameliorate the fitness cost of the host during evolution and promote plasmid survival.

Although this study highlights the importance of a cointegrate plasmid in AMR transmission, it still has some limitations. First, the structural diversity of the plasmid under different antibiotic pressures needs to be further explored. Second, the host species used in this study are limited. Third, the underlying mechanism leading to improved plasmid stability under positive selection still warrants further investigation. 

## 4. Conclusions

Overall, this study revealed that the cointegrate plasmid pSL131_IncA/C_IncX3, with high plasticity, could flexibly discard various regions and acquire other AMR genes in response to a complex environment, demonstrating its strong dissemination and evolution potential. Transferring this plasmid to clinic-associated strains could increase the biofilm formation ability, which would inevitably increase the treatment failure rates. The imposition of fitness cost could limit the spread of the cointegrate plasmid. However, the plasmid could stably maintain under high-risk pathogenic strain after exposure to antibiotic pressure, which emphasizes that antibiotic residue is an indispensable driving force in the development of MDR pathogens.

## Data Availability

The data presented in this study are openly available in Figshare at https://doi.org/10.6084/m9.figshare.14885238.v3 (accessed on 8 July 2021).

## Supplementary Materials

The following are available online at https://www.mdpi.com/article/10.3390/microorganisms9122435/s1, Figure S1: S1-PFGE of transconjugants from MR plates. Figure S2: S1-PFGE of transconjugants from MTR plates. Figure S3: Persistence of pSL131_IncA/C_IncX3-133K in three evolved clones after serial passaging in antibiotic-free medium. Figure S4: Circular comparison of cointegrate plasmid pSL131_IncA/C_IncX3 with two plasmid pSL131_IncA/C_IncX3-133K and pSL131_IncA/C_IncX3-ev1. Table S1: Basic information of pSL131_IncA/C_IncX3 with different structures. Table S2: Basic information of HS11286 and YZ6. Table S3: SNPs identified in three evolved strains as compared with the ancestral strain.

## References

[1] San Millan A., Pena-Miller R., Toll-Riera M., Halbert Z.V., McLean A.R., Cooper B.S., MacLean R.C. (2014). Positive selection and compensatory adaptation interact to stabilize non-transmissible plasmids. Nat. Commun..

[2] Carattoli A. (2013). Plasmids and the spread of resistance. Int. J. Med. Microbiol..

[3] Baltrus D.A. (2013). Exploring the costs of horizontal gene transfer. Trends Ecol. Evol..

[4] Harrison P.W., Lower R.P., Kim N.K., Young J.P. (2010). Introducing the bacterial ’chromid’: Not a chromosome, not a plasmid. Trends Microbiol..

[5] Carroll A.C., Wong A. (2018). Plasmid persistence: Costs, benefits, and the plasmid paradox. Can. J. Microbiol..

[6] Cottell J.L., Webber M.A., Piddock L.J. (2012). Persistence of transferable extended-spectrum-beta-lactamase resistance in the absence of antibiotic pressure. Antimicrob. Agents Chemother..

[7] Porse A., Schonning K., Munck C., Sommer M.O. (2016). Survival and Evolution of a Large Multidrug Resistance Plasmid in New Clinical Bacterial Hosts. Mol. Biol. Evol..

[8] Kamruzzaman M., Wu A.Y., Iredell J.R. (2021). Biological Functions of Type II Toxin-Antitoxin Systems in Bacteria. Microorganisms.

[9] Wu A.Y., Kamruzzaman M., Iredell J.R. (2020). Specialised functions of two common plasmid mediated toxin-antitoxin systems, ccdAB and pemIK, in *Enterobacteriaceae*. PLoS ONE.

[10] Xie M., Li R., Liu Z., Chan E.W.C., Chen S. (2018). Recombination of plasmids in a carbapenem-resistant NDM-5-producing clinical *Escherichia coli* isolate. J. Antimicrob. Chemother..

[11] Liu Z., Xiao X., Liu Y., Li R., Wang Z. (2020). Recombination of NDM-5-producing plasmids mediated by IS26 among *Escherichia coli*. Int. J. Antimicrob. Agents.

[12] Li R., Lu X., Peng K., Liu Z., Li Y., Liu Y., Xiao X., Wang Z. (2020). Deciphering the Structural Diversity and Classification of the Mobile Tigecycline Resistance Gene tet(X)-Bearing Plasmidome among Bacteria. Msystems.

[13] Li R., Lu X., Peng K., Liu Y., Xiao X., Wang Z. (2020). Reorganization of mcr-1-bearing large MDR plasmids resolved by nanopore sequencing. J. Antimicrob. Chemother..

[14] He D., Zhu Y., Li R., Pan Y., Liu J., Yuan L., Hu G. (2019). Emergence of a hybrid plasmid derived from IncN1-F33:A-:B- and mcr-1-bearing plasmids mediated by IS26. J. Antimicrob. Chemother..

[15] Chavda K.D., Chen L., Jacobs M.R., Rojtman A.D., Bonomo R.A., Kreiswirth B.N. (2015). Complete sequence of a bla(KPC)-harboring cointegrate plasmid isolated from *Escherichia coli*. Antimicrob. Agents Chemother..

[16] Liu P., Li P., Jiang X., Bi D., Xie Y., Tai C., Deng Z., Rajakumar K., Ou H.Y. (2012). Complete genome sequence of *Klebsiella pneumoniae* subsp. pneumoniae HS11286, a multidrug-resistant strain isolated from human sputum. J. Bacteriol..

[17] Li R., Xie M., Liu L., Huang Y., Wu X., Wang Z., Chan E.W.C., Chen S. (2020). Characterisation of a cointegrate plasmid harbouring blaNDM-1 in a clinical *Salmonella* Lomita strain. Int. J. Antimicrob. Agents.

[18] Zhao J., Zhang Y., Fan Y., Han J., Xiong Z., Liu X., Li B., Lu B., Cao B. (2021). Characterization of an NDM-5-producing hypervirulent *Klebsiella pneumoniae* sequence type 65 clone from a lung transplant recipient. Emerg. Microbes Infect..

[19] Li X., Mu X., Zhang P., Zhao D., Ji J., Quan J., Zhu Y., Yu Y. (2018). Detection and characterization of a clinical *Escherichia coli* ST3204 strain coproducing NDM-16 and MCR-1. Infect. Drug Resist..

[20] Hunter S.B., Vauterin P., Lambert-Fair M.A., Van Duyne M.S., Kubota K., Graves L., Wrigley D., Barrett T., Ribot E. (2005). Establishment of a universal size standard strain for use with the PulseNet standardized pulsed-field gel electrophoresis protocols: Converting the national databases to the new size standard. J. Clin. Microbiol..

[21] Bertani G. (2004). Lysogeny at mid-twentieth century: P1, P2, and other experimental systems. J. Bacteriol..

[22] Ma T., Fu J., Xie N., Ma S., Lei L., Zhai W., Shen Y., Sun C., Wang S., Shen Z. (2020). Fitness Cost of blaNDM-5-Carrying p3R-IncX3 Plasmids in Wild-Type NDM-Free *Enterobacteriaceae*. Microorganisms.

[23] De Gelder L., Ponciano J.M., Joyce P., Top E.M. (2007). Stability of a promiscuous plasmid in different hosts: No guarantee for a long-term relationship. Microbiology.

[24] Bankevich A., Nurk S., Antipov D., Gurevich A.A., Dvorkin M., Kulikov A.S., Lesin V.M., Nikolenko S.I., Pham S., Prjibelski A.D. (2012). SPAdes: A new genome assembly algorithm and its applications to single-cell sequencing. J. Comput. Biol..

[25] Seemann T. snippy: Fast Bacterial Variant Calling from NGS Reads. https://github.com/tseemann/snippy.

[26] Kolmogorov M., Yuan J., Lin Y., Pevzner P.A. (2019). Assembly of long, error-prone reads using repeat graphs. Nat. Biotechnol..

[27] Alikhan N.F., Petty N.K., Ben Zakour N.L., Beatson S.A. (2011). BLAST Ring Image Generator (BRIG): Simple prokaryote genome comparisons. BMC Genom..

[28] Sullivan M.J., Petty N.K., Beatson S.A. (2011). Easyfig: A genome comparison visualizer. Bioinformatics.

[29] Ni S., Li B., Tang K., Yao J., Wood T.K., Wang P., Wang X. (2021). Conjugative plasmid-encoded toxin-antitoxin system PrpT/PrpA directly controls plasmid copy number. Proc. Natl. Acad. Sci. USA.

[30] Partridge S.R., Kwong S.M., Firth N., Jensen S.O. (2018). Mobile Genetic Elements Associated with Antimicrobial Resistance. Clin. Microbiol. Rev..

[31] Yang X., Ye L., Chan E.W., Zhang R., Chen S. (2020). Tracking Recombination Events That Occur in Conjugative Virulence Plasmid p15WZ-82_Vir during the Transmission Process. Msystems.

[32] Zhao Q.Y., Li W., Cai R.M., Lu Y.W., Zhang Y., Cai P., Webber M.A., Jiang H.X. (2021). Mobilization of Tn1721-like structure harboring blaCTX-M-27 between P1-like bacteriophage in *Salmonella* and plasmids in *Escherichia coli* in China. Vet. Microbiol..

[33] Tang Y., Li G., Liang W., Shen P., Zhang Y., Jiang X. (2017). Translocation of Carbapenemase Gene blaKPC-2 both Internal and External to Transposons Occurs via Novel Structures of Tn1721 and Exhibits Distinct Movement Patterns. Antimicrob. Agents Chemother..

[34] Li G., Zhang Y., Bi D., Shen P., Ai F., Liu H., Tian Y., Ma Y., Wang B., Rajakumar K. (2015). First report of a clinical, multidrug-resistant *Enterobacteriaceae* isolate coharboring fosfomycin resistance gene fosA3 and carbapenemase gene blaKPC-2 on the same transposon, Tn1721. Antimicrob. Agents Chemother..

[35] Frech G., Schwarz S. (1999). Plasmid-encoded tetracycline resistance in *Salmonella enterica* subsp. enterica serovars choleraesuis and typhimurium: Identification of complete and truncated Tn1721 elements. FEMS Microbiol. Lett..

[36] Hua X., Zhang L., Moran R.A., Xu Q., Sun L., van Schaik W., Yu Y. (2020). Cointegration as a mechanism for the evolution of a KPC-producing multidrug resistance plasmid in Proteus mirabilis. Emerg. Microbes Infect..

[37] Lee H., Shin J., Chung Y.J., Park M., Kang K.J., Baek J.Y., Shin D., Chung D.R., Peck K.R., Song J.H. (2020). Co-introduction of plasmids harbouring the carbapenemase genes, blaNDM-1 and blaOXA-232, increases fitness and virulence of bacterial host. J. Biomed. Sci..

[38] Perez-Gallego M., Torrens G., Castillo-Vera J., Moya B., Zamorano L., Cabot G., Hultenby K., Alberti S., Mellroth P., Henriques-Normark B. (2016). Impact of AmpC Derepression on Fitness and Virulence: The Mechanism or the Pathway?. mBio.

[39] Costerton J.W., Stewart P.S., Greenberg E.P. (1999). Bacterial biofilms: A common cause of persistent infections. Science.

[40] Weingarten R.A., Johnson R.C., Conlan S., Ramsburg A.M., Dekker J.P., Lau A.F., Khil P., Odom R.T., Deming C., Park M. (2018). Genomic Analysis of Hospital Plumbing Reveals Diverse Reservoir of Bacterial Plasmids Conferring Carbapenem Resistance. MBio.

[41] Savage V.J., Chopra I., O’Neill A.J. (2013). Staphylococcus aureus biofilms promote horizontal transfer of antibiotic resistance. Antimicrob. Agents Chemother..

[42] Wein T., Hulter N.F., Mizrahi I., Dagan T. (2019). Emergence of plasmid stability under non-selective conditions maintains antibiotic resistance. Nat. Commun..

[43] Bouma J.E., Lenski R.E. (1988). Evolution of a bacteria/plasmid association. Nature.

[44] Harrison E., Guymer D., Spiers A.J., Paterson S., Brockhurst M.A. (2015). Parallel compensatory evolution stabilizes plasmids across the parasitism-mutualism continuum. Curr. Biol..

[45] Heuer H., Fox R.E., Top E.M. (2007). Frequent conjugative transfer accelerates adaptation of a broad-host-range plasmid to an unfavorable Pseudomonas putida host. FEMS Microbiol. Ecol..

[46] Knopp M., Andersson D.I. (2015). Amelioration of the Fitness Costs of Antibiotic Resistance Due To Reduced Outer Membrane Permeability by Upregulation of Alternative Porins. Mol. Biol. Evol..

[47] Bredin J., Saint N., Mallea M., De E., Molle G., Pages J.M., Simonet V. (2002). Alteration of pore properties of *Escherichia coli* OmpF induced by mutation of key residues in anti-loop 3 region. Biochem. J..

