# Supplementary material for: Structural Diversity, Fitness Cost, and Stability of a BlaNDM-1-Bearing Cointegrate Plasmid in Klebsiella pneumoniae and Escherichia coli
Source: Microorganisms. 2021 Nov 25;9(12):2435. doi: 10.3390/microorganisms9122435 (PMC8708245; doi:10.3390/microorganisms9122435)
Supplement: Supplementary file 1 [file microorganisms-09-02435-s001.zip › microorganisms-1460706-SI.pdf]

Supplementary Data

Structural diversity, fitness cost, and stability of a bla_{NDM-1}-bearing cointegrate plasmid in *Klebsiella pneumoniae* and *Escherichia coli*

Table S1. Basic information of pSL131_IncA/C_IncX3 with different structures.

Plasmids	Formation stage	Length	Replicons	The presence of TA or partitioning systems	Antibiotics resistance genes	Accession numbers
pSL131_IncA/C_IncX3	Original structure	216,896bp	IncA/C and IncX3	parA (IncX3) + parA (IncC) + parB + Rel-Xre-like +	*floR*, *tet*(A), *strAB*, *sul2*, *aph(3')-Ia*, *mph(A)*, *dfrA12*, *aadA2*, *sul1*, *bla*$_{NDM-1}$	MH105050
pMDRG14	After transferring the cointegrate plasmid into EC600 by conjugation	218,155bp	IncA/C and IncX3	parA (IncX3) + parA (IncC) + parB + Rel-Xre-like +	*floR*, *tet*(A), *strAB*, *sul2*, *aph(3')-Ia*, *mph(A)*, *dfrA12*, *aadA2*, *sul1*, *bla*$_{NDM-1}$	Online[1]
pMDRG21-157kb	After transferring the cointegrate plasmid into EC600 by conjugation	157,321bp	IncX3	parA (IncX3) + parA (IncC) + parB + Rel-Xre-like -	*floR*, *tet*(A), *strAB*, *sul2*, *dfrA12*, *aadA2*, *sul1*, *bla*$_{NDM-1}$	Online
pMDRG11-112kb	After transferring the cointegrate plasmid into EC600 by conjugation	112,151bp	IncX3	parA (IncX3) - parA (IncC) + parB + Rel-Xre-like -	*floR*, *tet*(A), *strAB*, *sul2*, *bla*$_{NDM-1}$	Online
pSL131_IncA/C_IncX3-133K	After transferring the cointegrate plasmid into YZ6 by conjugation	133,188bp	IncA/C and IncX3	parA (IncX3) + parA (IncC) + parB + Rel-Xre-like +	*floR*, *tet*(A), *strAB*, *sul2*, *bla*$_{NDM-1}$, *bla*$_{CTX-M-14}$	Online
pSL131_IncA/C_IncX3-ev1	Serial passaging under meropenem and tetracycline selection	133,487bp	IncA/C and IncX3	parA (IncX3) + parA (IncC) + parB + Rel-Xre-like +	*floR*, *tet*(A), *strAB*, *sul2*, *bla*$_{NDM-1}$, *bla*$_{CTX-M-14}$	Online

[1] The assembled plasmid sequences were deposited in Figshare database for reference. (https://doi.org/10.6084/m9.figshare.14885238.v3, accessed on 8 July 2021).

Table S2. Basic information of HS11286 and YZ6.

	Genotype type[1]	Source	Plasmids	Resistance genes
HS11286	ST11, $bla_{\text{KPC-2}}$	Clinical CRKP collected from sputum	pKPHS1 (~122kb)	$bla_{\text{CTX-M-14}}$
			pKPHS2 (~111kb)	$bla_{\text{KPC-2}}$, $bla_{\text{TEM-1C}}$
			pKPHS3 (~105kb)	*tet*(G), $bla_{\text{TEM-1C}}$, *aadA2*, *aac(3)-IId*, *sul2*, $bla_{\text{CTX-M-14}}$, *floR*
			pKPHS4 (3751bp)	N.D.[2]
			pKPHS5 (3353bp)	N.D.
			pKPHS6 (1308bp)	N.D.
YZ6	HS11286Δ$bla_{\text{KPC-2}}$ΔMDRΔKPHS_44780::Hm^{R}	Hygromycin resistant derivative of HS11286	pKPHS1 (~122kb)	$bla_{\text{CTX-M-14}}$
			pKPHS2 (~106kb)	$bla_{\text{TEM-1C}}$
			pKPHS4 (3751bp)	N.D.
			pKPHS5 (3353bp)	N.D.
			pKPHS6 (1308bp)	N.D.

[1] Hm^{R}, hygromycin resistance.

[2] N.D., not detected.

Table S3. SNPs identified in three evolved strains as compared with the ancestral strain.

Strain	SNP (amino acid substitution)	Nucleotide position	Product	Function
YZ6-pSL131_IncA/C_IncX3-ev1	G→A (-)[1]	43/1707	Outer membrane porin	Cell outer membrane<br>Integral component of membrane
	C→T (Arg282Lys)<br>G→A (Thr280Ile)<br>G→A (Thr273Ile)<br>T→C (-)	846/1266<br>840/1266<br>819/1266<br>489/1266	Outer membrane porin	Cell outer membrane<br>Integral component of membrane
	G→A (Arg118Gln)	354/510	Hypothetical protein	Unknown
	T→C (-)	Spacer		
	G→A (-)	Spacer		
	A→T (-)<br>T→G (Leu19*)<br>A→C (Gln22Pro)	43/204<br>57/204<br>66/204	Hypothetical protein	Unknown
YZ6-pSL131_IncA/C_IncX3-ev2	C→T (Thr272Met)<br>T→C (Ile277Thr)	816/1011<br>831/1011	Translation elongation factor Tu	Protein translation
	T→A (Ter400Leu)<br>G→A (Thr396Ile)<br>A→G (Ile393Thr)<br>T→C (Lys391Arg)<br>G→T (Ser371*)<br>C→T (Arg282Lys)<br>G→A (Thr280Ile)<br>G→A (Thr273Ile)<br>T→C (-)<br>G→A (Ser102Leu)<br>A→G (Leu52Pro)<br>A→G (Met47Thr)<br>A→G (Leu40Ser)	1200/1266<br>1188/1266<br>1179/1266<br>1173/1266<br>1113/1266<br>846/1266<br>840/1266<br>819/1266<br>489/1266<br>306/1266<br>156/1266<br>141/1266<br>120/1266	Outer membrane porin	Cell outer membrane<br>Integral component of membrane
	T→C (Leu40Ser)<br>T→G (Val54Glu)<br>C→T (Ala59Val)<br>C→T (Ala83Val)<br>G→A (Arg118Gln)	120/510<br>162/510<br>177/510<br>249/510<br>354/510	Hypothetical protein	Unknown
	G→A (-)	Spacer		
	A→T (-)<br>T→G (Leu19*)<br>A→C (Gln22Pro)	43/204<br>57/204<br>66/204	Hypothetical protein	Unknown

YZ6-pSL131_IncA/C_IncX3-ev3	G→A (Cys38Tyr)	114/543	Phage polarity suppression protein	Suppression of transcriptional polarity
	C→A (Ser45Tyr)	135/543		
	A→C (Gln132Pro)	396/543		
	G→T (Met143Ile)	430/543		
	C→T (Thr272Met)	816/1011	Translation elongation factor Tu	Protein translation
	T→C (Ile277Thr)	831/1011		
	C→C (Ser331Cys)	993/1266	Outer membrane porin	Cell outer membrane<br>Integral component of membrane
	G→A (Ala328Val)	984/1266		
	T→C (-)	489/1266		
	G→A (Ser102Leu)	306/1266		
	A→G (Leu52Pro)	156/1266		
	A→G (Met47Thr)	141/1266		
	A→G (Leu40Ser)	120/1266		
	T→A (Ile190Asn)	570/927	Branched-chain amino acid ABC transporter, permease protein LivH	Transmembrane transporter activity
	T→C (-)	Spacer		
	G→A (Arg118Gln)	354/510	Hypothetical protein	Unknown
	G→A (-)	Spacer		
	A→T (-)	43/204	Hypothetical protein	Unknown
	T→G (Leu19*)	57/204		
	A→C (Gln22Pro)	66/204		
	A→C (Gln132Pro)	396/543	Phage polarity suppression protein	Suppression of transcriptional polarity
	G→T (Met141Ile)	430/543		

[1] -, synonymous mutation.
*, including a stop codon.

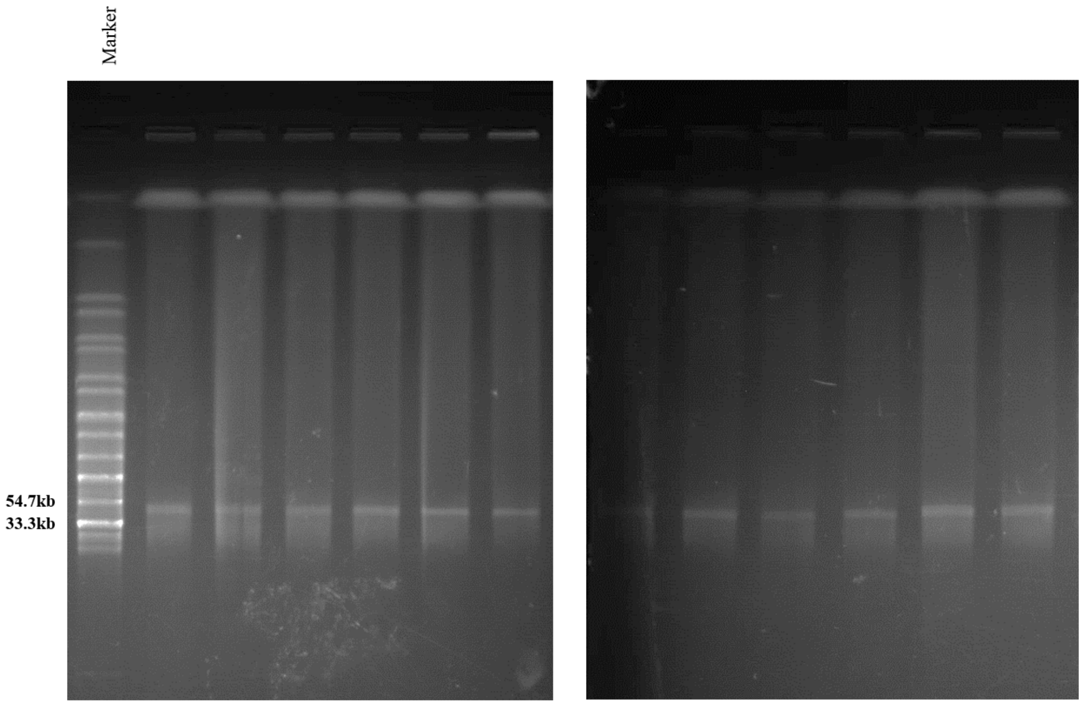

Figure S1. S1-PFGE of transconjugants from MR plates. The natural host SL131 of cointegrate plasmid was used as the donor strain, and EC600 was the recipient strain. Transconjugants were recovered from the plates supplemented with meropenem and rifampicin.

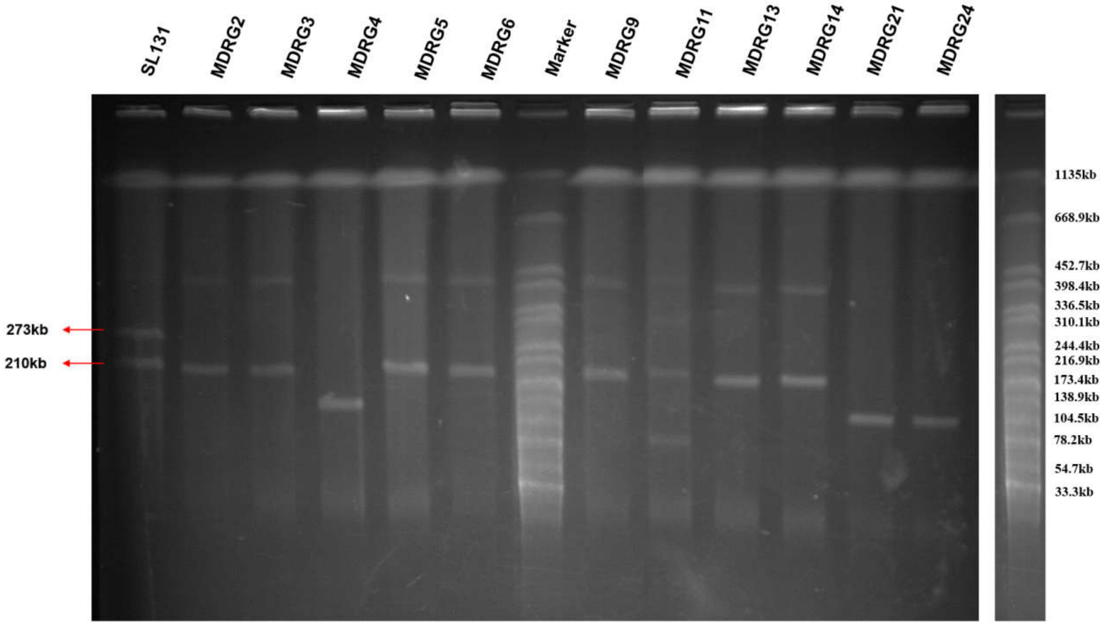

Figure S2. S1-PFGE of transconjugants from MTR plates. The natural host SL131 of cointegrate plasmid was used as the donor strain, and EC600 was the recipient strain. Transconjugants were recovered from the plates supplemented with meropenem, tetracycline, and rifampicin.

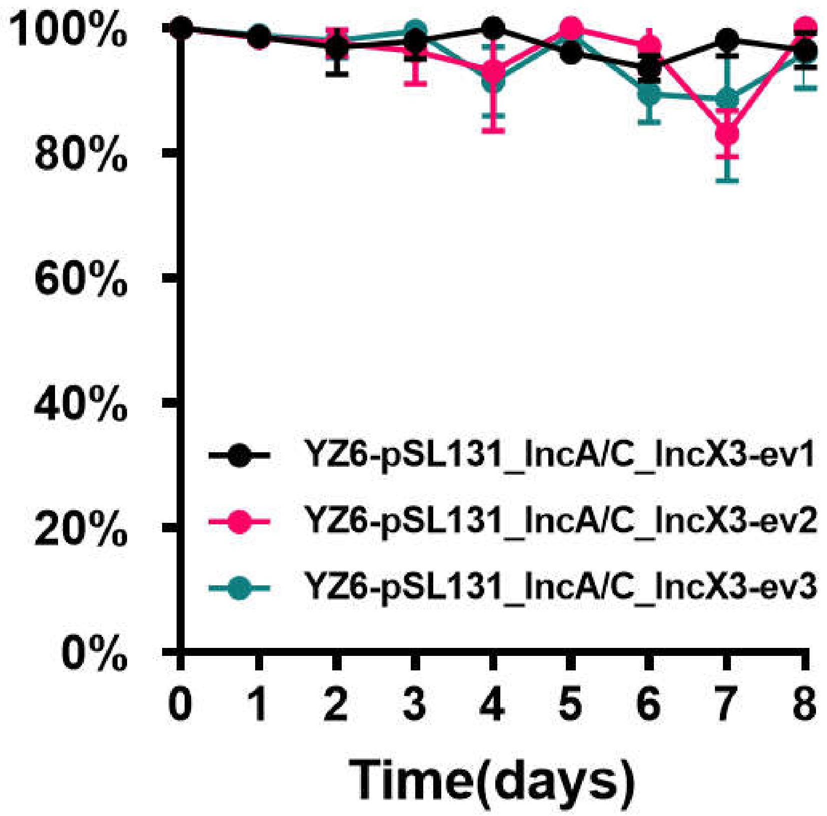

Figure S3. Persistence of pSL131_IncA/C_IncX3-133K in three evolved clones after serial passaging in antibiotic-free medium. Each point is the mean of 3 individual replicates and the error bars show the standard deviation.

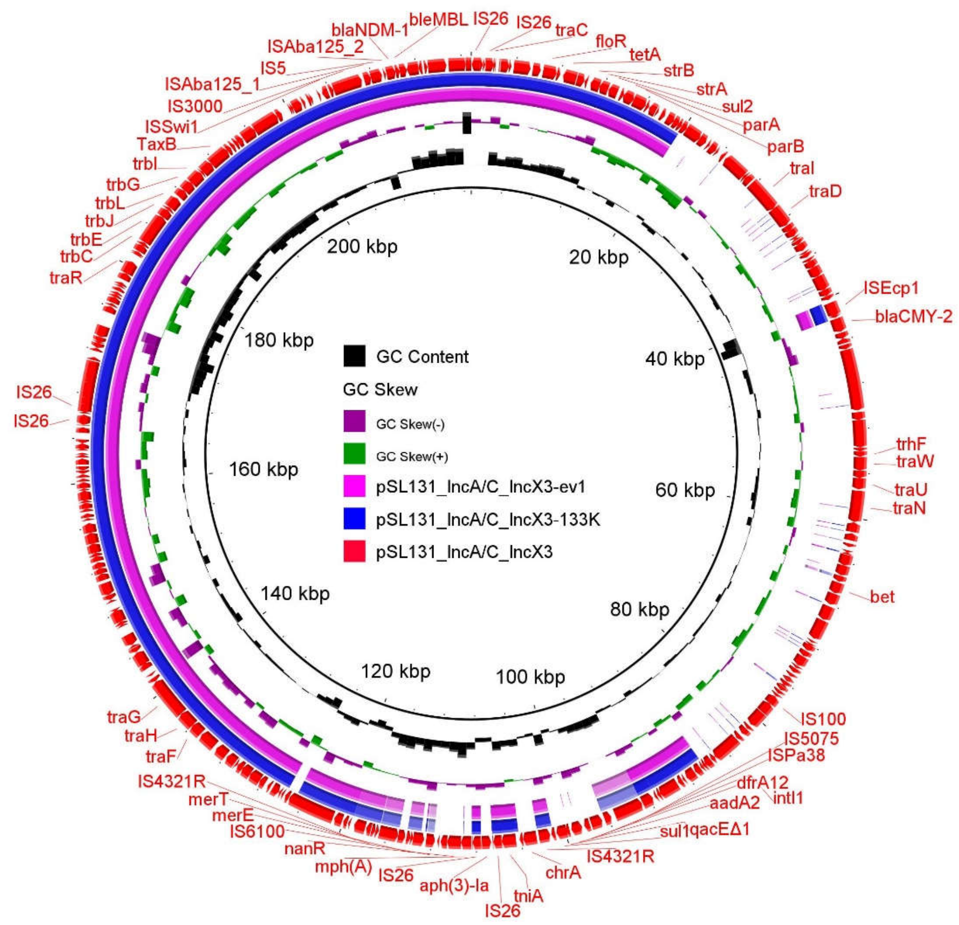

Figure S4. Circular comparison of cointegrate plasmid pSL131_IncA/C_IncX3 with two plasmid pSL131_IncA/C_IncX3-133K and pSL131_IncA/C_IncX3-ev1. Outmost circle with red arrows represents reference pSL131_IncA/C_IncX3.
